# T-Cell Clonal Expansion in Peripheral Blood Following Interventional Radiology Procedures for Metastatic Liver Cancer

**DOI:** 10.3390/cancers18091477

**Published:** 2026-05-04

**Authors:** Chenyang Zhan, Anita Karimi, Leila Haghani, Brett Marinelli, Wei Tian, Etay Ziv, Hooman Yarmohammadi, Erica S. Alexander, Vlasios S. Sotirchos, Ken Zhao, James W. Smithy, Alexander N. Shoushtari, David A. Scheinberg, Joseph P. Erinjeri

**Affiliations:** 1 Interventional Radiology Service, Department of Radiology, Memorial Sloan Kettering Cancer Center, 1275 York Avenue, New York, NY 10065, USA; zhanc@mskcc.org (C.Z.); sotirchv@mskcc.org (V.S.S.);; 2 Department of Medicine, Memorial Sloan Kettering Cancer Center, New York, NY 10065, USA; 3 Center for Experimental Therapeutics, Memorial Sloan Kettering Cancer Center, New York, NY 10065, USA

**Keywords:** embolization, ablation, metastatic liver cancer, T-cell clonality, TCR sequencing

## Abstract

Background: Interventional radiology (IR) procedures may induce anti-tumor immune responses, with subsequent clonal expansion of specific T-cells recognizing tumor antigens. This exploratory study aims to investigate the clonal expansion of T-cells in the peripheral blood after interventional radiology procedures for metastatic liver cancer. Methods: This prospective study included 16 patients with metastatic liver cancer undergoing IR procedures (13 embolizations and 3 ablations). Biopsy samples were collected prior to the procedure, and peripheral blood samples were obtained both pre- and post-procedure. Bulk T-cell receptor (TCR) sequencing was conducted to assess T-cell clonality dynamics and to evaluate the impact of procedures on the T-cell repertoire. Results: The overall clonality of the T-cell repertoire in the peripheral blood did not demonstrate significant variations following IR procedures (*p* = 0.27–0.77). A subset of patients exhibited limited T-cell clonal expansion in peripheral blood one month after IR procedures, with more than 10 T-cell clonotypes expanding in 43% of patients. Most clonal expansions decreased in frequency by three months; however, 3 patients displayed persistently expanding T-cell clonotypes in peripheral blood ≥ 3 months following IR procedures. Conclusions: IR procedures do not alter the overall clonality of the T-cell repertoire but may promote the expansion of a limited number of clonotypes. This study demonstrates that it is feasible to detect and longitudinally track expanding T-cell clonotypes following IR liver-directed therapy. Further studies are needed to determine whether these expanding clonotypes contribute to systemic anti-tumor immunity.

## Simple Summary

Interventional radiology (IR) procedures, such as ablation or embolization, are image-guided minimally invasive therapies that provide local tumor control and may also stimulate systemic anti-tumor immune response. This study investigated how these treatments to liver tumors affect T-cells by tracking their clonal expansion in peripheral blood. By sequencing T-cell receptors in 16 patients with metastatic liver cancer, this study found that while the overall T-cell clonality remained stable, a small subset of specific T-cells expanded following IR procedures. In some patients, these immune responses lasted for several months. These findings suggest that IR liver-directed therapy can induce a measurable T-cell clonal expansion, providing a framework for future studies to determine whether these expanding T-cell populations following IR therapies can improve patient outcomes, particularly when combined with other treatments.

## 1. Introduction

Immune checkpoint inhibitor (ICI) therapies enhance T-cell-mediated immune responses and have dramatically changed the management of oncologic patients. However, only a subset of patients demonstrated a long-term durable response [1,2]. One strategy to improve the response to immunotherapy is through combination with other treatment modalities, such as interventional radiology (IR) locoregional therapy [3,4,5,6,7]. Tumors treated by interventional radiology procedures, like transarterial embolization or thermal ablation, may cause increased presentation of tumor antigens and thus potentially prime the immune system to induce T-cell-mediated anti-tumor immunity, particularly when combined with immunotherapy [8,9,10,11]. However, there is a lack of understanding how IR procedures affect cell-mediated anti-tumor immunity. It remains unclear which locoregional treatment modalities or to which tumor types and sites may be most effective in generating an immunogenic response that can be synergistic with immunotherapy.

Cell-mediated anti-tumor immunity requires a T-cell receptor (TCR) on cytotoxic T-cell to recognize tumor antigen peptide presented by HLA-I complex on the tumor surface. The T-cell can be subsequently activated in the presence of co-stimulatory signals and cytokines to undergo clonal expansion and exert an adaptive immune response [12]. During T-cell development, the complementarity-determining region 3 (CDR3) of TCR undergoes recombination and nucleotide insertion/deletion steps to generate a diverse T-cell repertoire, allowing recognition of a wide range of pathogen antigens [13]. Each T-cell clonotype is defined by a unique TCR and the TCR of majority of T-cells comprise TCRα and TCRβ chains. The CDR3 region of the TCRβ chain is known to be responsible for the majority of antigen-binding specificity based on previous structural studies [14]. Therefore, next-generation sequencing of the TCRβ CDR3 region allows determination of T-cell clonal frequency, and monitoring of the dynamic changes in T-cell repertoire following systemic or locoregional treatments [15,16,17].

The liver is an immunotolerant organ [18]. There is a need to understand how liver-directed IR therapies influence systemic anti-tumor immunity [8,9,19]. If such therapies induce antigen-dependent antitumoral immune responses, this should be reflected by detectable T-cell clonal expansion following treatment. The clonality of T-cell repertoire can be determined by TCR sequencing; however, T-cell clonal dynamics following IR liver-directed therapies remain poorly defined. In this exploratory study, we used bulk TCR sequencing (TCRseq) to investigate T-cell clonal expansion in peripheral blood following IR procedures for metastatic liver cancer, and to evaluate the feasibility of longitudinally tracking T-cell clonality for future mechanistic and translational studies in metastatic liver cancer.

## 2. Materials and Methods

### 2.1. Study Cohort and Sample Collection

This IRB-approved prospective study comprised 16 patients with liver metastases (median age 68.5; 62.5% male, Table 1). A healthy donor (TCR28) with no active medical problems donated peripheral blood samples 1 month apart as a sham treatment. A patient with intrahepatic cholangiocarcinoma (ICC, TCR6) underwent a mapping angiogram, but the subsequent Y90 radioembolization was canceled due to a change in the treatment plan. The blood samples obtained before and after the mapping procedure for patient TCR6 were used as negative controls for T-cell clonal expansion.

The IR therapies included 3 percutaneous ablations and 13 embolization procedures. Eight patients underwent a percutaneous biopsy immediately before the IR therapeutic procedures, and samples were cryopreserved at −80 °C. Peripheral blood samples were acquired within 1 month prior (all 16 patients), and at both 1 month (14 patients) and 3 months (11 patients) post-procedure during standard-of-care blood work collection and were cryopreserved at −80 °C (Table 1, Figure S1). Three patients had blood sample collected at additional follow-up times for up to 6 months.

### 2.2. Procedures

The choice of locoregional therapy was based on multi-disciplinary team discussions, and technical details are outlined following. Hepatic arterial embolization (HAE) was performed according to a previously described study [20]. Briefly, the arteries to the target hepatic tumors were catheterized and embolized using Embospheres microspheres (40–120 µm, 100–300 µm, or a combination of them; Merit Medical Systems, South Jordan, UT, USA). Polyvinyl alcohol (PVA) (100 µm; Cook Medical, Bloomington, IN, USA) was also used in certain cases. The endpoint of embolization was complete stasis.

One patient (TCR31) received Y90 radioembolization with glass microspheres (TheraSphere^®^, Boston Scientific, Marlborough, MA, USA), and another patient (TCR34) was treated with resin microspheres (SIR-Spheres^®^; Sirtex Medical, Sydney, Australia). Dosimetry was determined using the MIRD method for both cases. TCR31 received TheraSphere microparticles with a dose of 116 Gy to the right hepatic lobe. TCR34 received SIR-Sphere microparticles with doses of 250 Gy to segment 2 and 200 Gy to segment 3.

One patient (TCR2) underwent cryoablation using Galil IcePearl (Boston Scientific, Marlborough, MA, USA). The cryoablation was performed with the first freeze cycle of 10 min and the first thaw cycle of 8 min, followed by the second freeze cycle of 10 min and a second passive thaw cycle. Another patient (TCR10) received microwave ablation to a liver tumor using an Emprint microwave probe (Medtronic, Mineola, NY, USA), with 10 min of ablation at 100 Watts at position 1 followed by 5 min of ablation at 100 Watts at position 2. A third patient (TCR13) received microwave ablation for a metastatic liver tumor using 2 Neuwave PR15 probes (Ethicon, Raritan, NJ, USA) at 60 Watts for 7 min.

### 2.3. TCRβ CDR3 Sequencing and Analysis

The fresh frozen biopsy samples and whole blood samples were delivered to Adaptive Biotechnologies for genomic DNA extraction. High-throughput sequencing of the TCRβ CDR3 region was performed using the Adaptive ImmunoSEQ platform (Adaptive Biotechnologies, Seattle, WA, USA) at the deep level as previously reported [16,21]. Non-productive sequences encoding a premature stop or frameshift were identified and excluded. T-cell clonal frequency was determined by dividing the number of a unique TCRβ rearrangement sequence by the total number of TCRβ sequences in the sample. The diversity of T-cell repertoire was evaluated using the Simpson clonality, defined as square root of the Simpson Dominance Index [22,23].

Since there can be baseline fluctuations in the T-cell repertoire, peripheral blood samples were collected 1 month apart from a healthy donor and a patient with ICC undergoing a diagnostic angiogram but no treatment as negative controls. Differential abundance analysis using the beta-binomial model [24] revealed multiple false positive T-cell clonal expansions in our negative control patients (Figure S2). Consequently, we established more stringent criteria for defining expanding T-cell clonotypes based on negative controls in this study, as follows: new T-cell clonotypes were defined as those undetected at baseline with a frequency above 0.025% at follow-up; increased T-cell clonotypes were defined as those with over a tenfold increase and a frequency above 0.01% at follow-up.

### 2.4. Statistical Analysis

The statistical analysis was performed using R (version 4.2.2, R core team, 2022). Clonality data were analyzed using the Shapiro test, indicating a non-normal distribution. Therefore, Simpson clonality comparisons between groups were analyzed using two-tailed Wilcoxon rank-sum tests. Tracking of T-cell clonotypes in longitudinal samples were performed using a custom R script. In all tests, *p* ≤ 0.05 was considered significant.

## 3. Results

This study investigated the impact on T-cell clonal dynamics following IR procedures for metastatic liver cancer in 16 patients. The cohort included eight patients with sarcoma liver metastases, and eight patients with melanoma liver metastases. Pre-procedure biopsy samples were collected from eight patients. Peripheral blood samples were collected at 1-month follow-up from 14 patients and at 3-month follow-up from 11 patients (Table 1). There were 13 embolotherapy procedures, including 11 hepatic artery embolizations (HAEs), and 2 Y90-radioembolizations (Y90). Three patients underwent ablation, including one cryoablation and two microwave ablations (MWAs). Three patients received systemic therapies within 1 month following IR procedures (NB003 for TCR11, and Nivolumab for TCR31 and 35), which represents a confounder in interpreting T-cell clonal expansion in these patients. In these cases, the observed clonal dynamics cannot be attributed solely to IR therapy.

### 3.1. T-Cell Repertoire Clonality

**Figure 1:**
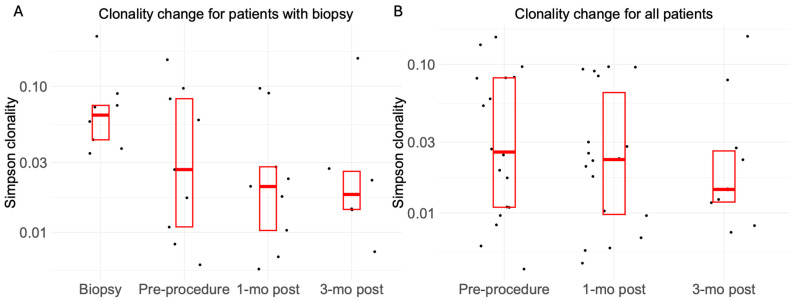
Simpson clonality for 8 patients who underwent biopsy (**A**) and for all patients in the study (**B**). Simpson clonality was used to assess the diversity of T-cell repertoire, with higher values indicating lower diversity and a more clonal repertoire. Each dot represents the clonality for an individual sample. Each box represents the 25–75th percentiles interquartile range (IQR), with the horizontal line inside each box indicating the median clonality value.

To assess the clonal diversity of the T-cell repertoire, we used the Simpson clonality index, with values ranging from 0 to 1. Values close to 1 represent monoclonal/oligoclonal samples, while values close to 0 represent polyclonal samples. All samples demonstrated low clonality (range 0.0042–0.2191). For patients with biopsies, the median Simpson clonality index for baseline biopsy samples (0.0635, range 0.0347–0.2191) was higher than baseline peripheral blood samples (0.0269, range 0.0060–0.1526), but this difference was not significant (*p* = 0.23) (Figure 1). For the overall cohort, peripheral blood T-cell clonality did not change significantly over time. Median clonality at baseline was 0.0258 (range 0.0042–0.1526), compared with 0.0229 at 1 month (range 0.046–0.0965; baseline vs. 1 month: *p* = 0.27) and 0.0140 at 3 months (range 0.0074–0.1549; baseline vs. 3 months: *p* = 0.77).

### 3.2. T-Cell Clonal Expansion

**Figure 2:**
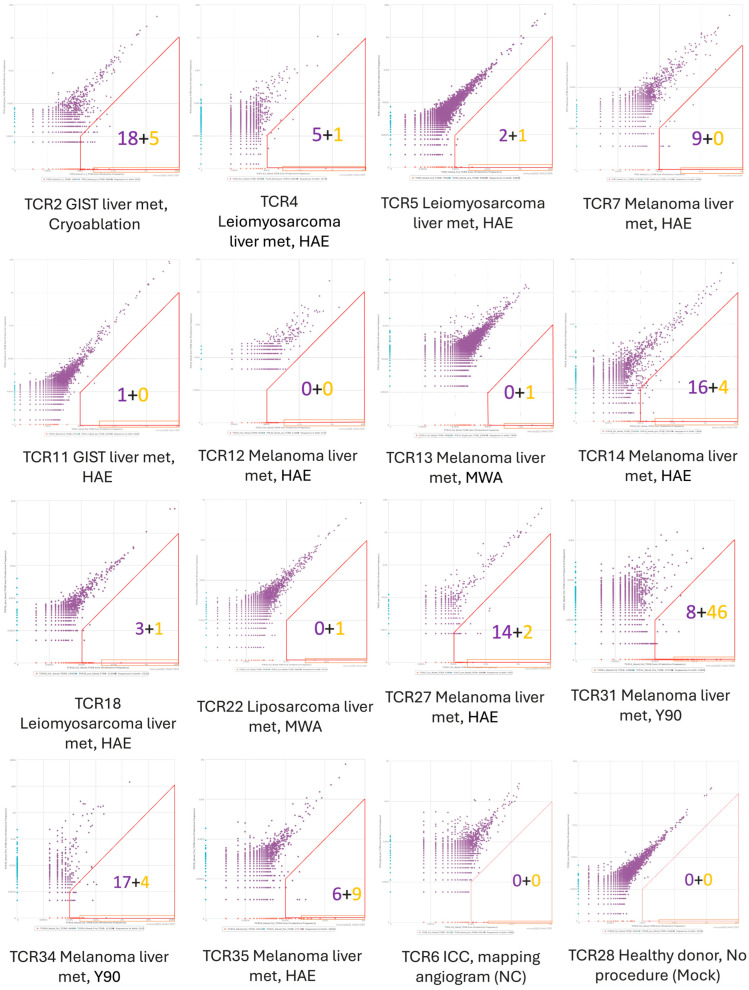
Pairwise comparison of T-cell clonal frequency before and 1 month after IR procedures. Each plot represents 1 patient. The *X*-axis indicates the percentage of T-cell clonal frequency at 1 month follow-up, while the *Y*-axis indicates the percentage of T-cell clonal frequency before the IR procedure. Criteria for expanded T-cell clonotypes post-IR therapies were determined based on comparison with negative controls (TCR28, healthy donor with no procedure and TCR6, a patient with intrahepatic cholangiocarcinoma who underwent a diagnostic mapping angiogram without IR therapy). Expanded T-cell clonotypes were defined as either non-detection at baseline with a frequency above 0.025% at follow-up (golden spots in golden rectangle boxes along *X*-axis, golden numbers), or as showing over a tenfold increase with a frequency above 0.01% at follow-up (purple spots in red trapezoid boxes, purple numbers).

**Figure 3:**
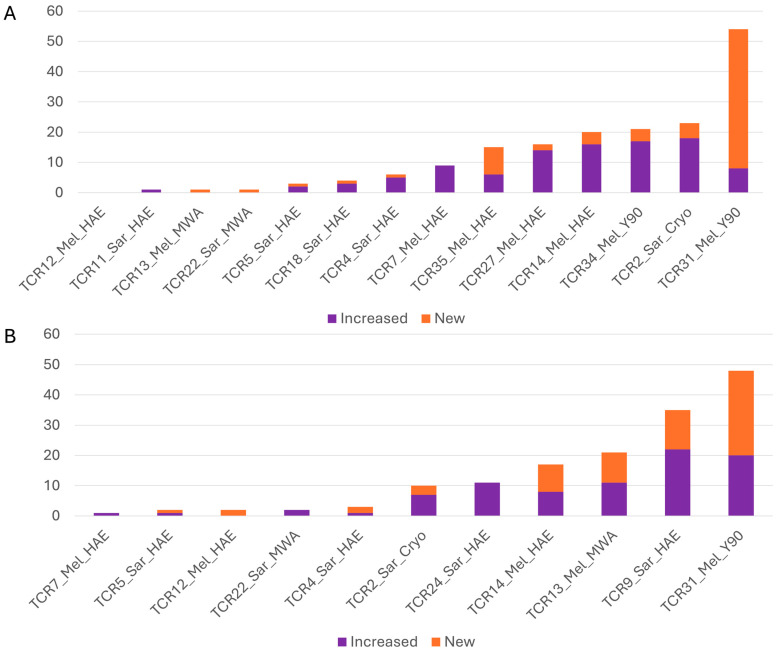
Histograms showing the expansion of T-cell clonotypes at 1-month (**A**) and 3-month (**B**) intervals following IR procedures. The patient IDs, tumor types, and procedures are labeled under the *X*-axis. Increased T-cell clonotypes are represented in purple, while new clonotypes are depicted in orange. Abbreviations: Mel, melanoma; Sar, sarcoma.

While the overall clonality in the T-cell repertoire did not change, there were expansion (new or increased frequency) of a limited number of T-cell clonotypes in peripheral blood of some patients following IR procedures (Figure 2 and Figure 3). One month post-procedure, the median number of expanding T-cell clonotypes in the peripheral blood was 7.5 (range: 0–54, IQR = 19, Figure 3A). In 43% of patients (6/14), over 10 expanding T-cell clonotypes were observed one month post-procedure. Three months post-procedure, the median number of expanding peripheral blood T-cell clonotypes was 10 (range: 1–48, IQR = 19, Figure 3B). In 55% of patients (6/11), 10 or more expanded T-cell clonotypes were observed three months post-procedure. When stratified by treatment modality, the median numbers of expanding clonotypes 1 month after treatment were 9 (IQR = 17) in the embolization group (HAE or Y90) and 1 (IQR = 22) in the ablation group (MWA or cryoablation). Given the limited sample size, particularly there were only three patients in the ablation group, these comparisons are descriptive and no formal statistical testing was performed.

### 3.3. Tracking of T-Cell Clonal Expansion

TCRseq analysis of baseline and longitudinal follow-up samples enabled tracking the frequency changes in each T-cell clonotype over the treatment course. Pre-procedure biopsy samples allowed for the identification of tumor-infiltrating lymphocytes (TILs), defined in this study as T-cell clonotypes detected in the biopsy samples. Here, we report in-depth analysis of the dynamics of T-cell clonal expansion in four most representative cases, selected based on clonal expansion and availability of longitudinal samples.

**Figure 4:**
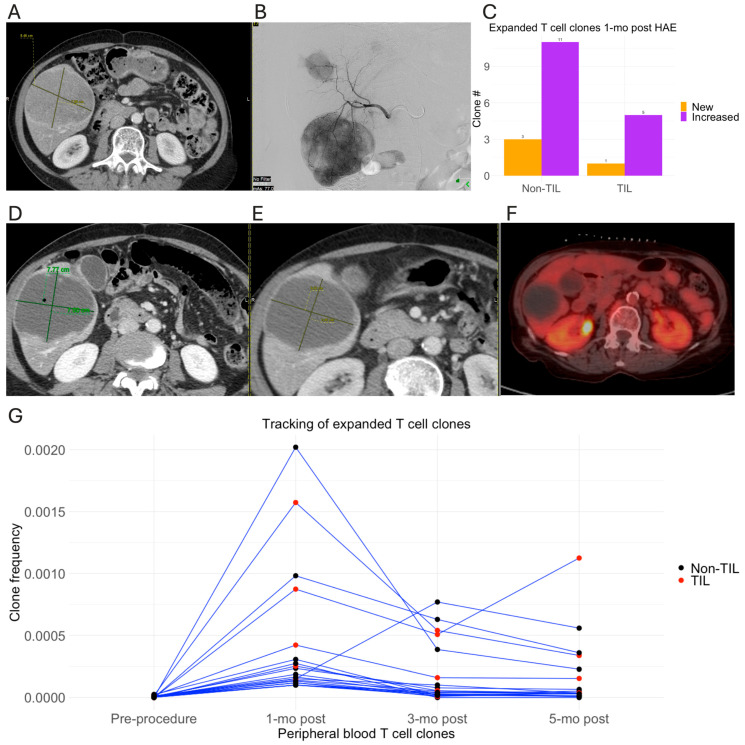
T-cell clonal expansion for a patient with metastatic uveal melanoma treated by HAE (TCR14). (**A**,**B**) Pre-procedure CT and intraprocedural angiogram showed multiple right hepatic lobe tumors with a dominant mass measuring 9 cm; (**C**) Histogram of increased (purple) and new (orange) TILs and non-TILs in the peripheral blood 1 month after procedure; (**D**) CT 1 month after HAE; (**E**) CT 3 months after HAE; (**F**) PET/CT 1 year after HAE; (**G**) frequency tracking of T-cell clonotypes expanded 1 month after HAE. The criteria used to define clonal expansion are detailed in the Methods section. Each line represents the clonal frequency of a single T-cell clonotype. TILs and non-TILS represented by red and black spots, respectively.

In Figure 4, we illustrate the case of a patient with metastatic uveal melanoma who experienced disease progression while undergoing immunotherapy (nivolumab+relatimab). Baseline CT imaging demonstrated multiple hepatic metastatic tumors, including a dominant mass measuring 9 cm (Figure 4A). The patient underwent HAE, targeting the tumors in the right lobe (Figure 4B), and continued with nivolumab+relatimab immunotherapy about 2 months post-HAE. Follow-up CT scans at 1 month and 3 months post-HAE, as well as PET/CT imaging 1-year post-HAE, showed complete necrosis of the treated liver tumors (Figure 4D–F). TCRseq analysis revealed expansion of T-cells clonotypes 1 month after the procedure, including 6 clonotypes of TILs and 14 clonotypes of non-TILs (Figure 4C). Seven of these clonotypes, including three TIL clonotypes, remain expanded 5 months after the procedure (Figure 4G).

**Figure 5:**
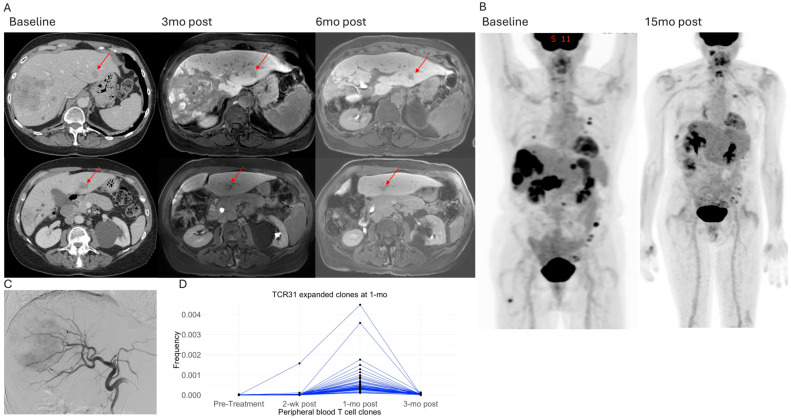
T-cell clonal expansion for a patient with metastatic uveal melanoma treated by Y90 (TCR31). (**A**) Baseline CT and follow-up MRI 3-month and 6-month after Y90. The red arrows annotate left lobe tumors (a segment 2 tumor in upper row, and a segment 3 tumor in lower row) not treated by Y90 which decreased size over time. (**B**) PET/CT imaging at baseline and 15 months after Y90 treatment demonstrated significantly decreased FDG uptake in the treated tumor within the right lobe. Additionally, the previously FDG-avid tumors in the left lobe and multiple extrahepatic sites at baseline showed no FDG avidity at the 15-month follow-up. (**C**) Angiogram at time of Y90 to the right lobe showed the dominant tumor. (**D**) Frequency tracking of T-cell clonotypes expanded 1 month after Y90-RE. Each line represents the clonal frequency of a single T-cell clonotype.

Figure 5 depicts a case of metastatic uveal melanoma in a patient presenting with a dominant right lobe tumor measuring 10cm and several small tumors in the left lobe (TCR31, Figure 5A). The patient underwent Y90 targeting the entire right lobe (Figure 5B,C). Subsequently, she started immunotherapy (nivolumab + relatimab), with the first dose 2 weeks after Y90. Follow-up MRI demonstrated good response to the treated right lobe tumor, atrophy of the right hepatic lobe, and compensatory hypertrophy of the untreated left lobe. Notably, over the 6 month follow-up, the sizes of the left lobe tumors not treated by Y90 were stable or slightly decreased (Figure 5A). Fifteen months after Y90, the FDG uptake in the treated right hepatic lobe was significantly reduced. The left hepatic lobe not treated by Y90, as well as multiple extrahepatic metastatic sites, demonstrated no discernible FDG uptake (Figure 5B). Two weeks post-Y90, five clonotypes of T-cells were expanded. One month post-Y90, there was expansion of 54 T-cell clonotypes. Since this patient received concurrent immunotherapy within 1 month of Y90, the T-cell clonal expansion observed at 1 month cannot be attributed solely to the IR procedure. None of these T-cell clonotypes had frequency meeting criteria for expansion 3 months after Y90 (Figure 5D).

**Figure 6:**
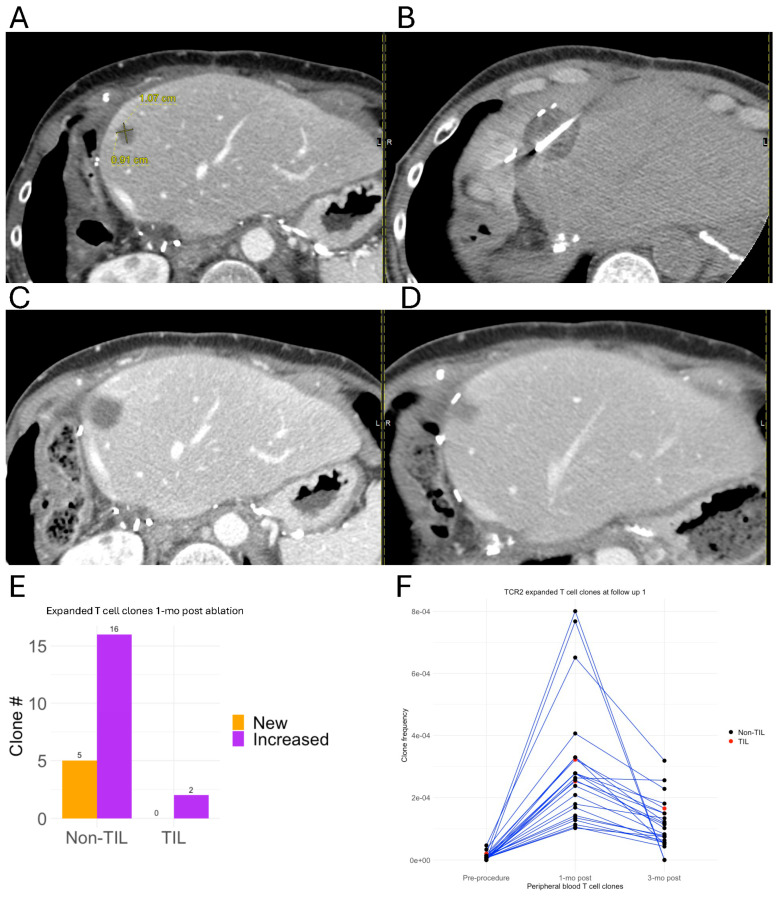
T-cell clonal expansion for a patient with metastatic GIST treated by cryoablation (TCR2). (**A**) Baseline CT (**B**) cryoablation (**C**,**D**) follow-up CT 1-month and 3-month after cryoablation. (**E**) Histogram of increased (purple) and new (orange) TILs and non-TILs in the peripheral blood 1 month after procedure. (**F**) Frequency tracking of T-cell clonotypes expanded 1 month after cryoablation. Each line represents a single T-cell clonotype. TILs and non-TILs represented by red and black spots, respectively.

A patient with metastatic GIST to the liver, status post-trisegmentectomy (TCR2), underwent cryoablation of a 1.1 cm tumor in the left lobe (Figure 6A,B). Follow-up CT at 1 month and 3 months post-ablation showed a decreased size of the ablated tumor (Figure 6C,D). TCRseq analysis at the 1-month follow-up demonstrated the expansion of 23 T-cell clonotypes, including 2 TIL clonotypes (Figure 6E,F). Six of these clonotypes, including one TIL clonotype, remained expanded at 3 months post-ablation (Figure 6F).

**Figure 7:**
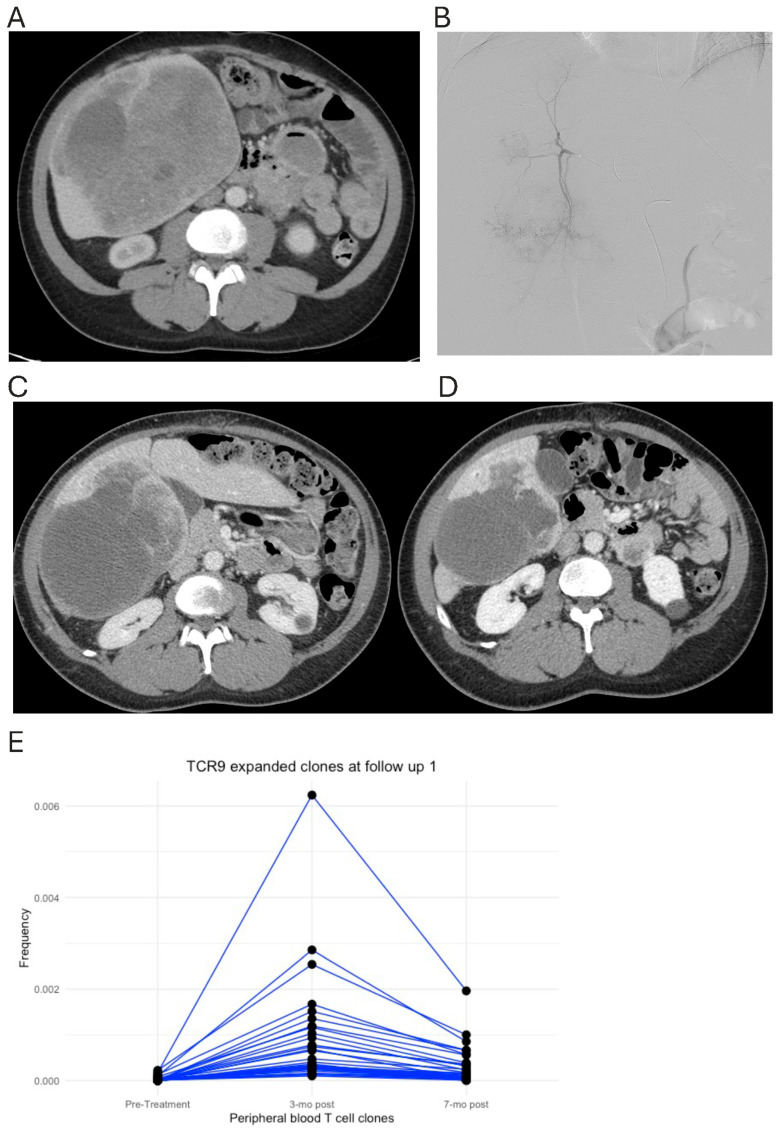
T-cell clonal expansion for a patient with metastatic GIST treated by HAE (TCR9). (**A**) Baseline CT (**B**) angiogram during HAE (**C**,**D**) follow-up CT 3 months and 7 months after HAE. (**E**) Frequency tracking of T-cell clonotypes expanded 3 months after HAE. Each line represents a T-cell clonotype.

In Figure 7, a patient with metastatic GIST (TCR9) underwent HAE to the dominant right hepatic lobe tumors (Figure 7A,B). Follow-up imaging showed decreased size and enhancement of the embolized tumor (Figure 7C,D). TCRseq showed 35 expanded T-cell clonotypes 3 months post-embolization, 6 of which remained expanded 7 months after the embolization (Figure 7E). No biopsy samples were collected for patients TCR9 and TCR31, so TILs could not be identified in these two patients.

## 4. Discussion

This exploratory study investigated the impact of IR procedures for metastatic liver cancer on T-cell clonality in peripheral blood, and demonstrated the feasibility to track dynamic changes in the clonal frequency of the expanding T-cells following IR procedures. Although the overall T-cell repertoire diversity in peripheral blood remained stable, a subset of patients exhibited an expansion of specific T-cell clonotypes following IR procedures.

Tracking T-cell clonal frequency in longitudinal samples revealed that a majority of T-cell clonotypes expanding at the 1-month follow-up decreased in frequency by the 3-month follow-up, although some clonotypes still met the criteria for expansion compared to pre-procedure samples at 3-month follow-up. This likely correlates with a decreased inflammatory response in the treated tumor over time after the procedure. Notably, some TIL clonotypes showed expansion in peripheral blood 1 month after the procedure. The proliferation of these TIL clonotypes after IR procedures may result from activation stimulated by released tumor antigens. However, the majority of the expanding T-cell clonotypes were non-TILs. These non-TILs which expanded after IR procedures may represent T-cells stimulated by new tumor antigens released after the procedure, TILs not initially detected by TCRseq in the biopsy sample due to sampling bias or low frequency, or T-cells undergoing bystander activation rather than specific recognition of tumor antigens [25]. It is unclear whether such limited clonal expansion following liver-directed therapies may lead to clinically relevant systemic immune response.

In a study investigating TIL clonality changes following cryoablation in combination with immunotherapy for early-stage breast cancer, Page et al. reported a greater expansion of intratumoral and peripheral blood T-cell clonotypes in patients who received both cryoablation and ipilimumab compared to either therapy alone (9). In this study, cryoablation of the primary breast tumor alone did not induce a discernible T-cell clonal expansion in peripheral blood, possibly due to the short interval between cryoablation and follow-up blood collection (median 7 days, range 4–10 days). In contrast, our study observed T-cell clonal expansion in peripheral blood one month after the IR liver-directed therapies. For blood collected 2 weeks following procedure, fewer T-cell clonal expansions were detected (Figure 5D). In renal cell carcinoma patients treated by external beam radiotherapy, Chow et al. reported an expansion of TIL clonotypes in the peripheral blood 2 weeks following radiotherapy, with a subsequent contraction to baseline levels by week 4 [26]. These findings are in line with our results, which showed a similar trend in T-cell clonal dynamics—expanding at 1 month and contracting at 3 months, although the timing of the dynamics vary. In a study investigating the combination of radiation therapy and CTLA-4 blockade for metastatic non-small-cell lung cancer, Formenti et al. reported that the patients who responded to therapy exhibited significantly larger number of T-cell clonal expansions in peripheral blood 22 days post-radiation [15]. Of note, in this study, two neoantigen-specific CD8+ T-cell clonotypes in a patient with complete response were found to be persistently expanded in the peripheral blood up to 200 days after radiotherapy. Similarly, in our study, three patients demonstrated persistently expanded T-cell clonotypes in peripheral blood for 3 months or more after the IR procedure (Figure 4G, Figure 6F and Figure 7E). Notably, to our knowledge, this is the first report of systemic T-cell clonality changes induced by locoregional therapies in an immunotolerant organ.

Bulk TCR sequencing utilized in this study enables quantification of T-cell clonal frequency across a large number of T-cell clonotypes. Clonotype frequency determined by TCR sequencing may represent a valuable biomarker to dissect the mechanism of systemic response of local therapies. However, this sequencing approach focuses on a small segment of the CDR3 loop within the TCRβ gene, thereby providing no information about the T-cell phenotype or subtype. Notably, the specific antigen peptides recognized by these TCRs remain unknown, raising uncertainly about whether the expanded T-cells recognize tumor antigens. Current methods for predicting TCR specificity are constrained by the limited availability of known TCR-binding MHC-restricted peptides, and do not perform well when only one CDR sequence is known [27,28]. A more practical approach would be to adopt a “reverse immunology” strategy [29], similar to the method reported by Formenti et al. This approach involves predicting immunogenic MHC-I-restricted tumor antigens based on genomic sequencing of the tumor sample, followed by identification and tracking of tumor-specific T-cell clonal frequency using TCR sequencing in the peripheral blood [15]. Metastatic melanoma represents an ideal model using reverse immunology approach to investigate tumor-reactive T-cell clonal expansion following IR therapy. This is attributed to its well-characterized tumor-associated antigens, including gp100, PRAME, and NY-ESO-1; its relatively high mutation burden, which generates a larger pool of tumor-specific antigens; and evidence from this study demonstrating the presence of expanding T-cell clonotypes in the peripheral blood of patients with metastatic melanoma after IR procedures.

This exploratory study is limited by a relatively small sample size, a heterogeneous patient population, and varied procedure types. This exploratory design is not powered for subgroup analysis stratified by patient or tumor characteristics. The absence of detectable T-cell clonal expansion in some patients likely reflect variations in tumor biology and different liver-directed therapies. Given the lack of prior studies investigating T-cell clonal dynamics following liver-directed therapies, this approach was intended to broadly assess the feasibility of detecting T-cell clonal expansion as a measure of systemic immune responses. Due to the exploratory nature of this study, correlation analyses between T-cell clonal expansion and clinical biomarkers or outcomes were not possible. The feasibility of longitudinally tracking T-cell clonal frequency demonstrated in this study enables future investigations in a larger and more homogenous cohort, such as metastatic melanoma, to better define the impact of liver-directed therapy on systemic T-cell clonal expansion, the association with other biomarkers, and its predictive value in clinical outcome.

As discussed above, the results in this study did not identify the antigens recognized by the limited clonal expansion and it is not known whether the responses are tumor-specific. The phenotype of the expanding T-cell clonotypes is also undefined, precluding the determination of whether they represent effector populations (e.g., cytotoxic T-cells) or immunosuppressive populations (e.g., regulatory T cells). Additional immune profiling approaches can be used to characterize the underlying immune response, including analysis of T-cell subsets by flow cytometry or single-cell RNA sequencing [30,31], along with profiling of plasma cytokines. Integration of such complementary analysis with bulk TCR sequencing will be important to elucidate the mechanisms underlying immune responses to liver-directed IR therapies, particularly within the immunotolerant hepatic microenvironment. Furthermore, it remains critical to ascertain whether the observed expansion of T-cell clonotypes can translate into therapeutic benefits, and if so, to explore strategies to harness this induced systemic antitumoral immunity, including optimal timing to combine with immunotherapy. While this study does not directly determine whether IR liver-directed therapy induces antitumoral immunity, it provides a methodological framework to address this question in future translational studies.

## 5. Conclusions

In conclusion, interventional radiology liver-directed therapies do not cause significant change in clonality in the T-cell repertoire, but may induce limited T-cell clonal expansion. This exploratory study demonstrates the feasibility of identifying and tracking expanding T-cell clonotypes following IR procedures, which may serve as potential biomarkers or therapeutic targets. Further studies are needed to characterize their antigen specificity, phenotype, and therapeutic potential to guide the combination of IR therapies with systemic immunotherapy.

## Figures and Tables

**Table 1:** Patient Demographics and Sample Collections. Abbreviations: HAE, hepatic artery embolization; MWA, microwave ablation; Y90, Y90 radioembolization. A checkmark indicates that a sample was collected at the corresponding time point.

Patient	Gender	Age	Cancer	Treatment	Biopsy	Pre-Procedure Blood	1-Month Follow-Up Blood	3-Month Follow-Up Blood
TCR2	Female	60	GIST liver met	Cryoablation	**√**	**√**	**√**	**√**
TCR4	Male	67	Leiomyosarcoma liver met	HAE	**√**	**√**	**√**	**√**
TCR5	Female	77	Leiomyosarcoma liver met	HAE		**√**	**√**	**√**
TCR6	Male	70	ICC	Mapping		**√**	**√**	
TCR7	Male	66	Melanoma liver met	HAE	**√**	**√**	**√**	**√**
TCR9	Male	48	GIST liver met	HAE		**√**		**√**
TCR11	Male	51	GIST liver met	HAE		**√**	**√**	
TCR12	Male	84	Melanoma liver met	HAE	**√**	**√**	**√**	**√**
TCR13	Female	34	Melanoma liver met	MWA	**√**	**√**	**√**	**√**
TCR14	Female	75	Melanoma liver met	HAE	**√**	**√**	**√**	**√**
TCR18	Male	57	Leiomyosarcoma liver met	HAE		**√**	**√**	
TCR22	Male	56	Liposarcoma liver met	MWA		**√**	**√**	**√**
TCR24	Female	76	GIST liver met	HAE		**√**		**√**
TCR27	Male	90	Melanoma liver met	HAE	**√**	**√**	**√**	
TCR28	Male	41	None	Control		**√**	**√**	
TCR31	Female	76	Melanoma liver met	Y90		**√**	**√**	**√**
TCR34	Male	81	Melanoma liver met	Y90	**√**	**√**	**√**	
TCR35	Male	77	Melanoma liver met	HAE		**√**	**√**	

## Data Availability

The TCR sequencing data generated in this study are not publicly available due to patient privacy and ethical restrictions.

## Supplementary Materials

The following supporting information can be downloaded at: https://www.mdpi.com/article/10.3390/cancers18091477/s1, Figure S1: Flow diagram of peripheral blood sample availability. Persistent T-cell expansions were observed in TCR2 (3 months), TCR14 (5 months) and TCR9 (7 months). Figure S2: Differential abundance analysis using beta-binomial model showed multiple false positive expansion of T-cell clonotypes (blue circles) based on negative controls in the study (TCR6 and TCR28).

## Abbreviations

The following abbreviations are used in this manuscript:

TCR	T-cell receptor
HAE	hepatic artery embolization
MWA	microwave ablation
Y90	Y90 radioembolization
Mel	Melanoma
Sar	Sarcoma
