# Supplementary material for: T-Cell Clonal Expansion in Peripheral Blood Following Interventional Radiology Procedures for Metastatic Liver Cancer
Source: Cancers (Basel). 2026 May 4;18(9):1477. doi: 10.3390/cancers18091477 (PMC13162843; doi:10.3390/cancers18091477)
Supplement: Supplementary file 1 [file cancers-18-01477-s001.zip › cancers-4257649-supplementary.pdf]

Supplementary Materials:

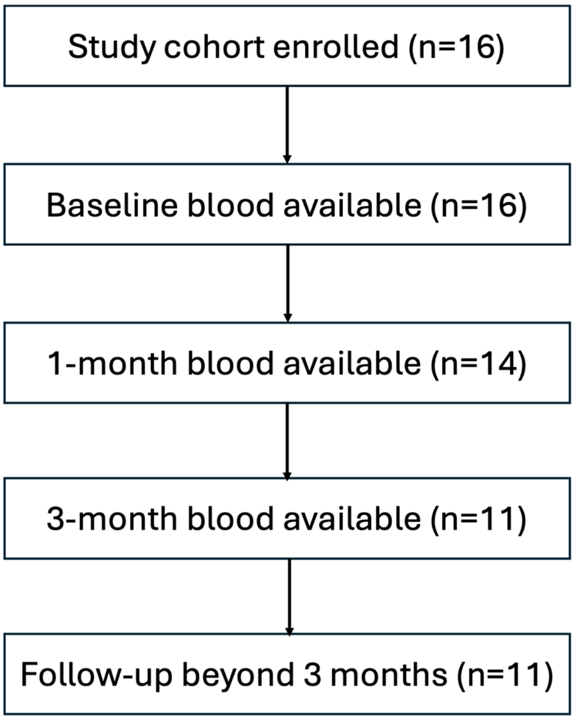

Figure S1: Flow diagram of peripheral blood sample availability. Persistent T-cell expansions were observed in TCR2 (3 months), TCR14 (5 months) and TCR9 (7 months).

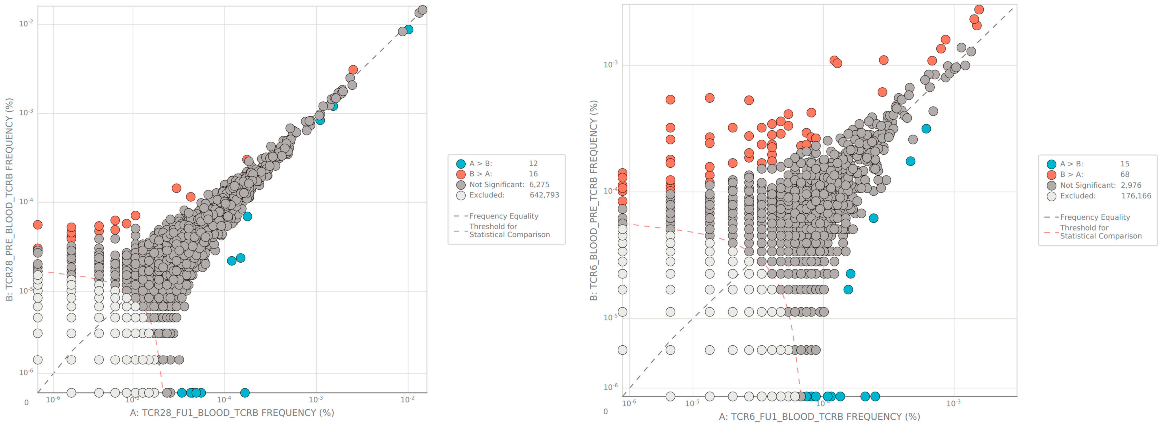

Figure S2: Differential abundance analysis using beta-binomial model showed multiple false positive expansion of T-cell clonotypes (blue circles) based on negative controls in the study (TCR6 and TCR28).
